# Inflammation-induced PINCH expression leads to actin depolymerization and mitochondrial mislocalization in neurons

**DOI:** 10.1186/s40035-020-00211-4

**Published:** 2020-08-03

**Authors:** Kalimuthusamy Natarajaseenivasan, Santhanam Shanmughapriya, Prema Velusamy, Matthew Sayre, Alvaro Garcia, Nestor Mas Gomez, Dianne Langford

**Affiliations:** 1 Department of Neurosciences and Center for Neurovirology, Lewis Katz School of Medicine at Temple University, Philadelphia, PA 19140 USA; 2 Department of Microbiology, Bharathidasan University, Tiruchirappalli, 620024 India; 3 Heart and Vascular Institute, Department of Medicine, Department of Cellular and Molecular Physiology, Penn State College of Medicine, Hershey, PA 17033 USA

**Keywords:** Neuron, Mitochondria, Actin, PINCH, Neuroinflammation

## Abstract

**Background:**

Diseases and disorders with a chronic neuroinflammatory component are often linked with changes in brain metabolism. Among neurodegenerative disorders, people living with human immunodeficiency virus (HIV) and Alzheimer’s disease (AD) are particularly vulnerable to metabolic disturbances, but the mechanistic connections of inflammation, neurodegeneration and bioenergetic deficits in the central nervous system (CNS) are poorly defined. The particularly interesting new cysteine histidine-rich-protein (PINCH) is nearly undetectable in healthy mature neurons, but is robustly expressed in tauopathy-associated neurodegenerative diseases including HIV infection and AD. Although robust PINCH expression has been reported in neurons in the brains of patients with HIV and AD, the molecular mechanisms and cellular consequences of increased PINCH expression in CNS disease remain largely unknown.

**Methods:**

We investigated the regulatory mechanisms responsible for PINCH protein-mediated changes in bioenergetics, mitochondrial subcellular localization and bioenergetic deficits in neurons exposed to physiological levels of TNFα or the HIV protein Tat. Changes in the PINCH-ILK-Parvin (PIP) complex association with cofilin and TESK1 were assessed to identify factors responsible for actin depolymerization and mitochondrial mislocalization. Lentiviral and pharmacological inhibition experiments were conducted to confirm PINCH specificity and to reinstate proper protein-protein complex communication.

**Results:**

We identified MEF2A as the PINCH transcription factor in neuroinflammation and determined the biological consequences of increased PINCH in neurons. TNFα-mediated activation of MEF2A via increased cellular calcium induced PINCH, leading to disruption of the PIP ternary complex, cofilin activation by TESK1 inactivation, and actin depolymerization. The disruption of actin led to perinuclear mislocalization of mitochondria by destabilizing the kinesin-dependent mitochondrial transport machinery, resulting in impaired neuronal metabolism. Blocking TNFα-induced PINCH expression preserved mitochondrial localization and maintained metabolic functioning.

**Conclusions:**

This study reported for the first time the mechanistic and biological consequences of PINCH expression in CNS neurons in diseases with a chronic neuroinflammation component. Our findings point to the maintenance of PINCH at normal physiological levels as a potential new therapeutic target for neurodegenerative diseases with impaired metabolisms.

## Background

Particularly interesting new cysteine-histidine-rich protein (PINCH) is highly expressed during development, but data from our laboratory have shown that PINCH is nearly undetectable in healthy adult brains [1–4]. However, PINCH is robustly expressed in the brains of patients with neuroinflammatory or neurodegenerative disease with a tauopathy component including human immunodeficiency virus (HIV) infection [1, 3, 4], Alzheimer’s disease (AD) [3], and epilepsy [2]. In vitro, PINCH is induced in neurons by exposure to tumor necrosis factor-α (TNFα) directly, or indirectly by exposure to the HIV transactivator of transcription (Tat) protein that triggers TNFα production [1, 3, 5–7]. Studies have shown that PINCH facilitates the formation and accumulation of hyperphosphorylated Tau (hpTau) via AKT/GSK3β signaling; whereas blocking TNFα-induced PINCH expression with TNF-receptor antagonist or with siRNA or shRNA against PINCH reduces the formation and accumulation of hpTau in neurons [1, 3].

PINCH is a non-catalytic protein consisting of five double-zinc-finger LIM domains that bind to several different proteins including integrin-linked kinase (ILK) and Nck-2 [8]. In addition to interacting with PINCH, ILK also interacts with Parvin to form the PINCH-ILK-Parvin (PIP) complex [9–11]. The functions of the PIP complex are cell type-dependent. Studies utilizing dominant negative mutants of PINCH, ILK, and Parvin that inhibit protein-protein interactions provide evidence that the PINCH, ILK and Parvin complex formation is critical for cell adhesion, spreading, motility, and extracellular matrix (ECM) assembly [10–15]. Furthermore, genetic studies in *C. elegans* and *Drosophila* show that mutations of gene homologs of *PINCH* [16, 17], *ILK* [18, 19], or *Parvin* [20] disrupt assembly of integrin-actin complexes and cell-ECM attachment. The PIP complex is also important for the regulation of cell adhesion, cytoarchitecture and apoptosis [21]. While the functions of the PIP complex have been extensively studied in non-neuronal cells, much less is known about the role of PIP in the central nervous system (CNS).

In this context, the ECM plays important roles in guiding molecules during cell migration in CNS development, and is implicated in the maintenance of stable neuronal connections and regulation of synaptic plasticity [22, 23]. At the molecular level, cellular adhesion with the ECM is mediated by a network of transmembrane adhesion receptors, integrins and integrin-proximal cytoplasmic proteins. The cytoplasmic components, PINCH, ILK and Parvin, link integrins to the actin cytoskeleton and to signaling proteins [24–26]. In fact, there are several brain disorders that are characterized by mutations in actin-regulatory proteins [22, 27, 28].

Given the fundamental role of PINCH in the PIP complex and the altered expression of PINCH under neuroinflammatory conditions, PINCH is highly likely to contribute to neurodegenerative processes in CNS diseases with neuroinflammation. The regulatory mechanisms responsible for the induction of PINCH and the role of the PIP complex in neurodegenerative diseases are unknown. This prompted us to investigate whether inflammation-induced PINCH expression alters PIP complex formation and functioning in neurons. We report here for the first time, that surrogate models of neuroinflammation (TNFα- or Tat-treated neurons) induce PINCH by calcium-dependent transcriptional regulation. The increased expression of PINCH in neurons led to disassociation of parvin from the PIP complex, promoting parvin’s interaction with and inactivation of testicular protein kinase 1 (TESK1) [24, 29–33], an actin-associated kinase. The inactivation of TESK1 prevented TESK1-mediated cofilin phosphorylation, thereby increasing the actin depolymerization activity of cofilin [34–36]. The depolymerization of actin disrupted the tubulin-kinesin-miro complex, causing mislocalization of mitochondria to perinuclear regions of the neuron. Our results provide new information on the cause and consequences of induced PINCH expression in neuroinflammatory conditions. Furthermore, the integrity of the PIP complex is essential for neurons to maintain actin stabilization and mitochondrial distribution.

## Materials and methods

### Human primary neurons

Fetal brain tissues (gestational age, 16–18 weeks) for isolation of neurons were obtained from elective abortion procedures performed in full compliance with ethical guidelines of National Institutes of Health and Temple University. Neurons were provided by the Comprehensive NeuroAIDS Center at Temple University. Briefly, the fetal brain tissue was washed with cold Hanks-balanced salt solution (HBSS), and meninges and blood vessels were removed. The tissue in HBSS was digested with papain (0.8 mg/ml) for 30 min at 37 °C, washed with HBSS, resuspended in NM5 media (neurobasal media supplemented with 5% horse serum, 1% B27, 1% glutamax, and gentamycin), and further dissociated by repeated pipetting to obtain single-cell suspensions. The cell suspension was passed through a 70-μm cell strainer and cells were counted. The single-cell suspension was plated in a poly-*D*-lysine-coated 60-mm dish at a density of ~ 3.0 × 10^6^ cells/dish in NM5 media. Twenty-four hours later, the culture medium was completely replaced with neurobasal media without horse serum (NM0). Four days later, half of the medium was removed and replaced with neurobasal media (NM0) supplemented with 1.25 μM 5-Fluoro-2′-deoxyuridine (FDU) and 1.5 μM uridine. The purity of cultures was assessed by immunolabeling with antibodies for MAP2, GFAP and Iba-1 and results showed over 99% of neuronal labeling (MAP2), while Iba-1 and GFAP were undetectable.

### Treatment conditions

After 14 days in culture, the neurons were treated with 50 ng/ml of recombinant Tat (rTat, 101 amino acids, ImmunoDX LLC, Woburn, WA) or 50 ng/ml of recombinant human TNFα (Sigma Aldrich, St. Louis, MO) for 48 h. The concentrations used were based on results from previous publications that utilized primary neurons to evaluate cell toxicity and PINCH expression [1, 3]. Neurons not exposed to Tat or TNFα served as controls.

### Plasmids and lentivirus

Light Switch Promoter Reporter GoClone plasmid with *lims1/pinch* (PINCH) (NM_004987) promoter sequence (SwitchGear Genomics; S712264) and the corresponding control plasmids were used for the luciferase assay. The ready-to-use human CACNA1G siRNA lentivirus (Applied Biological Materials Inc. Richmond, BC, Canada) was used to knock down Cav3.1 in human primary neurons. Mission® lentiviral transduction particles (TRCN0000365202) and Mission® TRC3 Human ORF lentivirus particles (TRCN0000468941) were used to knock down and overexpress PINCH, respectively. PINCH knockdown (KD) and overexpression were confirmed by Western blotting 72 h post-infection.

### Quantitative measurement of PINCH expression

Neurons treated with or without Tat/TNFα for 48 h were harvested and total RNA was isolated using RNeasy Mini Kit (Qiagen). cDNA was generated using an iScript™ cDNA Synthesis Kit (Bio-Rad, USA). Semi-quantitative real-time PCR was performed using SYBR green reagents (LightCycler®96, Roche, USA) using PINCH-specific primers: forward 5′-GCCTGTTCTACCTGCAACAC-3′, reverse 5′-CCTTCCTAAGGTCTCAGCTAGT-3′. GAPDH was used as a loading control.

### Western blotting

Cell extracts were prepared from neurons treated with or without Tat/TNFα using RIPA buffer (containing 50 mM Tris-HCl, pH 7.4, 150 mM NaCl, 0.25% deoxycholic acid, 1 mM EDTA, 1% NP-40, and protease and phosphatase inhibitor cocktail, Thermo Scientific). Protein concentrations were quantified using the Pierce™ 660 nm protein assay reagent. Equal amounts of protein samples (25 μg/well) were separated on a 4%–12% Bis-Tris polyacrylamide gel, transferred to a nitrocellulose membrane using iBlot 2 NC regular stacks (Thermo Scientific), and probed with antibodies specific for Ca^2+^/calmodulin-dependent protein kinase II (CAMKII) (1:1000, Abcam), phospho-CAMKII (1:1000, Abcam), pP38 (1:1000, Cell Signaling), P38 (1:1000, Cell Signaling), pMEF2A (1:1000, Abcam), MEF2A (1:1000, LifeSpan BioSciences), PINCH (1:1000, BD Biosciences), GAPDH (1:5000, Santa Cruz), ILK1 (1:1000, Abcam), Parvin (1:1000, Cell Signaling), TESK1 (1:1000, Cell Signaling), pCofilin at serine 3 (1:1000, Cell Signaling), Cofilin (1:1000, Cell Signaling), Chronophin (1:1000, Cell Signaling), Tubulin (1:1000, Cell Signaling), mitochondrial Rho GTPase 1 (Miro1) (1:1000, Abcam), KIF5B, kinesin (1:1000, Abcam), or Trak1 (1:1000, ThermoFisher).

### ChIP, quantitative PCR, and luciferase activity

The ChIP assay was performed using a Pierce™ Magnetic ChIP kit. In brief, DNA-protein complexes from neurons exposed to Tat or TNFα were crosslinked and immunoprecipitated using ChIP-validated antibodies against RNA polymerase II, MEF2A, c-Jun, Foxd1, HOXA9, or negative control IgG. Immune complexes were extracted and analyzed by quantitative PCR using primers (5′-TTCAGGCAAGGACACCCTCA-3′ and 3′-TCCGACTGAGTCACCTCCTGG-5′) that flank specific regions in the PINCH promoter. Values were normalized to input DNA. Results were depicted as the fold enrichment over basal expression. Neurons were transfected with luciferase reporter plasmids (4 μg) containing the PINCH promoter sequence with binding elements for MEF2A. After 48 h, neurons were lysed, and the luciferase activity was measured with LightSwitch Luciferase Assay Reagent using a plate reader (BiotTek Cytation1).

### Measurement of cytosolic Ca^2+^ (cCa^2+^)

Human primary neurons grown on 50 μg/ml poly-*D*-lysine (Sigma Aldrich, St Louis)-coated 35-mm glass coverslips were exposed to Tat or TNFα for 48 h. After treatment, the neurons were loaded with 5 μM Fluo-4/AM for 30 min in extracellular medium at room temperature as previously described [37]. Coverslips were mounted in an open perfusion microincubator (PDMI-2; Harvard Apparatus) at 37 °C and imaged. Confocal images were acquired at 488 nm excitation using a 63× oil objective (LSM 800; Carl Zeiss, Inc.). Images were analyzed and Ca^2+^ fluorescence quantified by using ImageJ (NIH).

### Immunofluorescence labeling of PINCH and actin

Human primary neurons grown on 50 μg/ml poly-*D*-lysine (Sigma Aldrich)-coated 35-mm glass coverslips were exposed to Tat or TNFα for 48 h. After treatment, the neurons were fixed in PBS containing 4 (*w*/*v*) paraformaldehyde for 20 min at room temperature, permeabilized with 0.5% (*v*/*v*) Triton X-100 for 15 min at room temperature and then blocked in PBS containing 3% bovine serum albumin for 1 h. The cells were then incubated with anti-PINCH antibody (1:200 dilution; BD Biosciences) overnight at 4 °C. After 3 washes with 1× PBS, the neurons were incubated for 1 h with Alexa Fluor 488-conjugated anti-mouse IgG (Invitrogen; 1:200 dilution) and rhodamine phalloidin (1:1000, Invitrogen) to simultaneously label PINCH and actin, respectively. After 1-h incubation, neurons were washed with PBS and mounted with Vectashield containing DAPI (Vector Lab., Burlingame, CA, USA). Confocal images were obtained at 405 nm (DAPI), 488 nm (PINCH), and 561 nm (actin) excitations respectively using a 63× oil objective (LSM 800; Carl Zeiss, Inc.). The length of actin filaments was manually measured with ImageJ software (NIH). All of the images taken had a resolution of 0.592 pixels/μm. Using measure analysis tool in ImageJ we measured the pixel length of each filament in an image.

### Analysis of neuronal distribution of mitochondria

Human primary neurons grown on 50 μg/ml poly-*D*-lysine (Sigma Aldrich)-coated 35-mm glass coverslips were exposed to Tat or TNFα for 48 h. After treatment, the neurons were loaded with Rhodamine 123 (1 μM) for 20 min at 37 °C and confocal images were obtained at 488 nm using a 63× oil objective (LSM 800; Carl Zeiss, Inc.). The changes in mitochondrial distribution were assessed and compared with untreated control cells. The distance of mitochondria from neuronal nucleus was measured using ImageJ.

### Co-immunoprecipitation and Western blot analysis

Protein extracts were collected from neurons treated with or without Tat or TNFα using RIPA buffer (50 mM Tris-HCl, pH 7.4, 150 mM NaCl, 0.25% deoxycholic acid, 1 mM EDTA, 1% NP-40, protease and phosphatase inhibitor cocktail, Thermo Scientific). The extracts were immunopurified with antibodies specific for kinesin or PINCH or Parvin and Western blotted with antibodies specific for tubulin, Miro1, kinesin, TRAK1, ILK, PINCH, Parvin, and TESK1.

### TNFα ELISA

A human TNFα ELISA Kit was used to quantify TNFα in culture media of neurons exposed to Tat for different lengths of time according to the manufacturer’s protocol (ThermoFisher Scientific).

### Mitochondrial oxygen consumption rate (OCR)

Non-targeting (control) shRNA and PINCH KD neurons (3 × 10^5^) were exposed to Tat or TNFα for 48 h. OCR was measured at 37 °C in an XF96 extracellular flux analyzer (Seahorse Bioscience). Neurons were sequentially challenged with 2 μM oligomycin, 0.5 μM FCCP, and 0.5 μM rotenone plus antimycin A to measure basal and maximal OCR, ATP-coupled respiration, spare capacity, and proton leak.

### Statistical analyses

Data from multiple experiments (*n* ≥ 3) are expressed as mean ± SEM, and differences between groups were analyzed using two-tailed paired Student’s *t*-test or, when not normally distributed, a nonparametric Mann-Whitney U test. Differences in means among multiple datasets were analyzed using one-way ANOVA with the Kruskal-Wallis test, followed by pairwise comparison using the Dunn test. *P* ≤ 0.05 was considered as statistically significant in all analyses. The data were computed either with GraphPad Prism version 7.0 or SigmaPlot 11.0 software.

## Results

### PINCH is transcriptionally regulated by MEF2A under inflammatory conditions

**Fig. 1:**
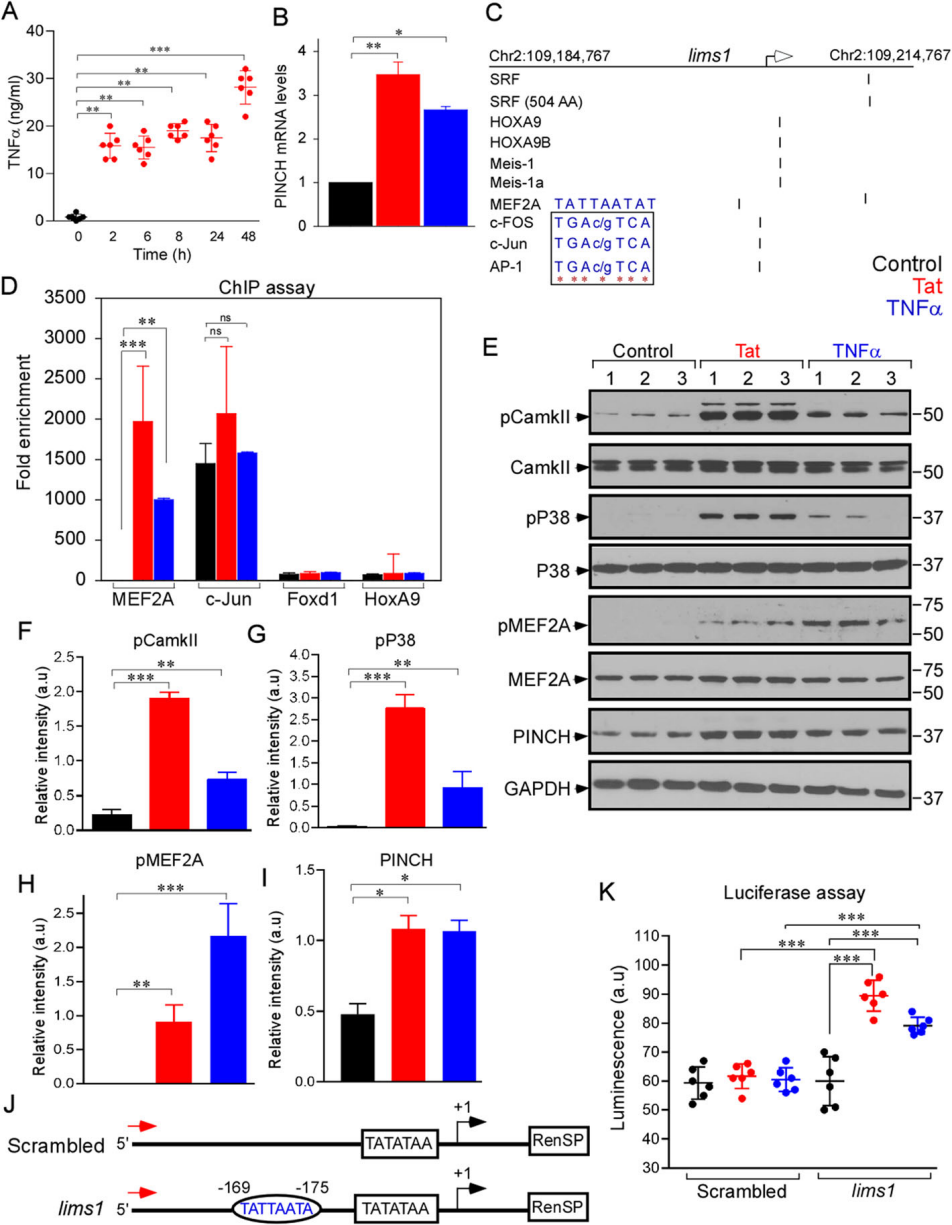
PINCH is transcriptionally regulated by MEF2A under inflammatory conditions. **a** Quantification of TNFα production by neurons exposed to Tat at different time points. **b** Quantification of PINCH mRNA levels in neurons untreated or exposed to Tat or TNFα for 48 h. **c** Bioinformatic analysis of *lims1/pinch* promoter sequence predicted a conserved putative binding site for different transcription factors (TF). Inset: Sequences show conserved binding sites for MEF2A, Cc-FOS, Cc-Jun, and AP-1. **d** ChIP-assay was performed in neurons untreated or exposed to Tat or TNFα. Antibodies specific for MEF2A, c-Jun, Foxd1, and HoxA9 were used to immunoprecipitate the chromatin and the fold enrichment of *lims1/pinch* promoter relative to the matched input control was quantified by qPCR. **e** Representative Western blot of lysates from neurons untreated or exposed to Tat or TNFα and probed with antibodies specific for phospho-CamkII, CamkII, phospho-P38, P38, phospho-MEF2A, MEF2A, PINCH and GAPDH. **f-i** Quantification of relative protein abundance of phospho-CamkII/CamkII (**f**), phospho-P38/P38 (**g**), phospho-MEF2A/MEF2A (**h**), and PINCH/GAPDH (**i**) from (**e**). **j** Schematic representation of the *lims1/pinch* promoter-luciferase constructs. The MEF2A consensus response element at − 169-175 base pairs (bp) (TATTATA) is shown in the oval. **k** Neurons transfected with control and *lims1/pinch* luciferase constructs were untreated or exposed to Tat or TNFα for 48 h and luciferase activity was measured. Data represent mean ± SEM; *P < 0.05; **P < 0.01; ***P < 0.001; *n* = 3–5 (one-way ANOVA)

Since Tat induces TNFα in a feed forward loop [1, 3, 5–7, 38, 39] (Fig. 1a), we exposed human primary neurons to Tat or TNFα to investigate the mechanisms of PINCH induction. Similar to increased protein expression [1, 3], a significant increase in PINCH mRNA levels (Fig. 1b) was observed in neurons exposed to Tat or TNFα, indicating the transcriptional regulation of PINCH by these inflammatory factors. We next examined potential transcription factors responsible for PINCH transcription.

Bioinformatics analysis of the PINCH (*lims1/pinch*) promoter sequence predicted conserved putative binding sites for several transcription factors, including MEF2A, c-FOS, c-Jun, and AP-1 upstream of the *lims1/pinch* transcription start site (Fig. 1c). To identify the transcription factor(s) that regulate PINCH expression in neuroinflammatory conditions, we performed a ChIP assay after exposure to Tat or TNFα. Neurons exposed to Tat or TNFα showed significantly increased MEF2A binding to the *lims1/pinch* promoter (Fig. 1d), indicating MEF2A as a potential transcriptional activator of PINCH. c-Jun also showed binding, but the binding was not regulated in response to Tat or TNFα exposure. As P38 is a potent kinase that activates MEF2A [40–43], we next assessed changes in the P38-dependent cascade for MEF2A activation by Western analysis. Consistent with the increased MEF2A binding to *lims1/pinch* promoter, Western blotting analysis showed increased phosphorylation of MEF2A (Fig. 1e, h) in neurons exposed to Tat or TNFα. Data also suggested that MEF2A phosphorylation was mediated through the CAMKII signaling pathway (Fig. 1e-h). To further confirm MEF2A as an activator for PINCH expression, we generated luciferase reporter constructs of the *lims1/pinch* promoter with an MEF2A binding site (Fig. 1j). Neurons expressing the luciferase construct were stimulated with Tat or TNFα, and luciferase activity was measured. The neurons expressing the *lims1/pinch* promoter construct (− 169–175 bp) showed increased luciferase activity in response to Tat or TNFα (Fig. 1k), indicating MEF2A-mediated transcriptional regulation of PINCH during neuroinflammatory conditions.

### Elevated cCa^2+^ activates MEF2A through P38 phosphorylation and facilitates PINCH expression

Because increased cCa^2+^ activates the calcium/calmodulin-dependent kinase [44], we next asked if increased phosphorylation of CAMKII and the downstream signaling cascade for PINCH expression is the result of elevated cCa^2+^ in neurons exposed to Tat or TNFα. Measurement of cCa^2+^ using Fluo-4-AM showed significantly increased Ca^2+^ fluorescence in neurons exposed to Tat or TNFα (Supplementary Fig. S1A). As the low-voltage-activated T-type Ca^2+^ channel Cav3.1 is involved in triggering αCaMKII activation [45], we transiently knocked down Cav3.1 in neurons and as expected, the transient KD of Cav3.1 (Supplementary Fig. S1C, D) in neurons normalized the elevated cCa^2+^ even after exposure to Tat or TNFα (Supplementary Fig. S1B). Then we assessed the expression of PINCH and tested if the relative upstream cascade was disrupted in Cav3.1 KD neurons exposed to Tat or TNFα. In line with decreased cCa^2+^ levels, phosphorylation of P38 and MEF2A was significantly decreased (Supplementary Fig. S1C, E, F), accompanied by decreased PINCH expression (Supplementary Fig. S1C, G). To further confirm that the decreased activation of MEF2A in Cav3.1 KD neurons blocked the Tat- or TNFα-induced PINCH expression, we performed ChIP and luciferase assays. Consistent with decreased phosphorylation of MEF2A, the binding of MEF2A to *lims1/pinch/pinch* promoter was decreased in Tat- or TNFα-treated Cav3.1 KD neurons (Supplementary Fig. S1H). These results were further confirmed by decreased luciferase activity in Cav3.1 KD neurons exposed to Tat or TNFα (Supplementary Fig. S1I). Taken together, these data show that under conditions of neuroinflammation, PINCH expression is transcriptionally regulated by MEF2A through a cCa^2+^-dependent regulatory cascade (Supplementary Fig. S2).

### Increased PINCH expression disrupts the PIP complex and is accompanied by actin disassembly

**Fig. 2:**
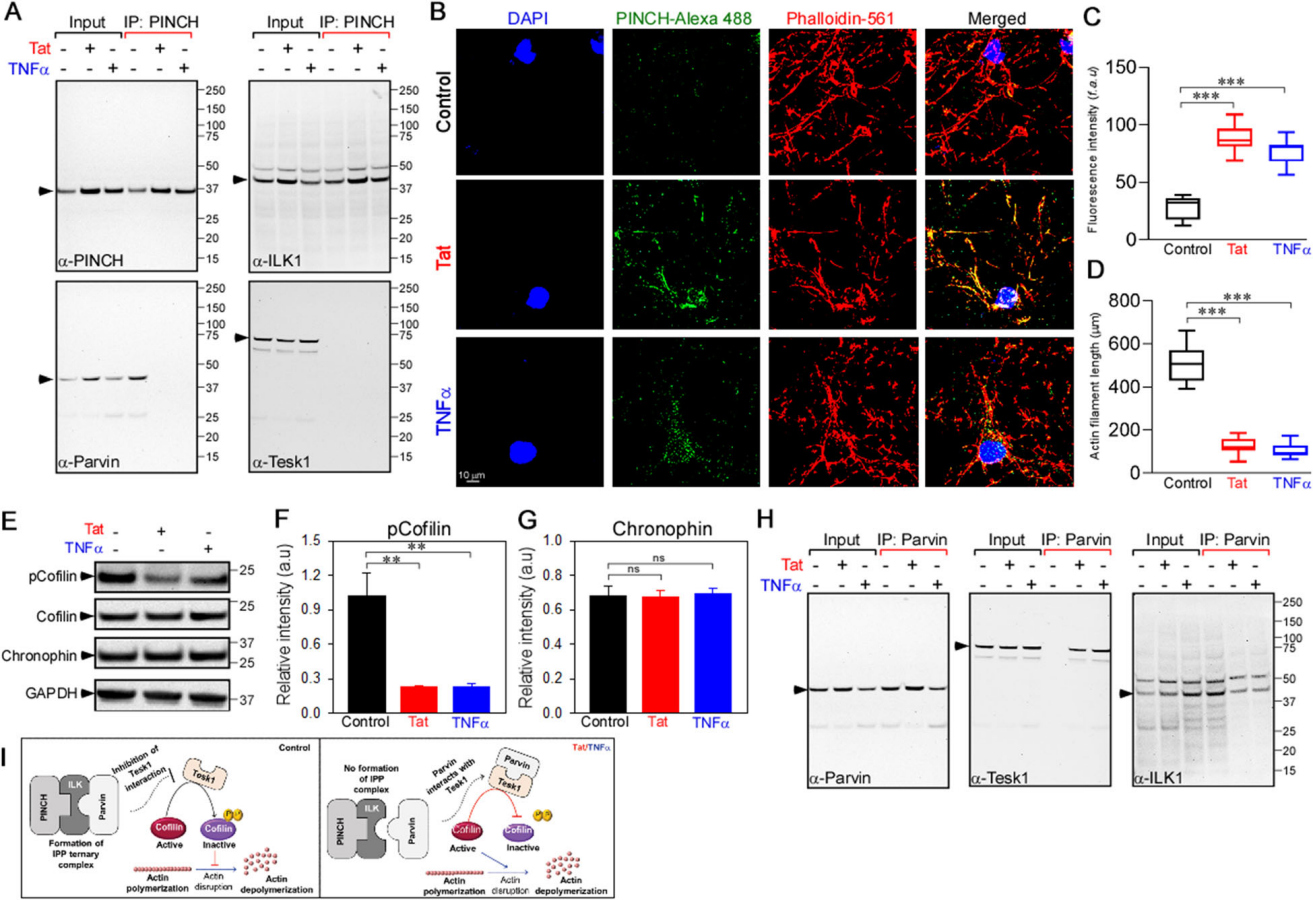
Increased PINCH expression disrupts the PIP complex and is accompanied by actin disassembly. **a** Cell lysates from control, Tat- or TNFα-treated neurons were immunoprecipitated with anti-PINCH antibody. Following immunoprecipitation, total cell lysates (input) and immunoprecipitated materials (IP) were subjected to Western blot analysis. Samples were probed with antibodies specific for PINCH (top left), ILK1 (top right), α-Parvin (bottom left), and TESK1 (bottom right). **b** Changes in cellular actin cytoarchitecture were observed using confocal microscopy in neurons untreated or exposed to Tat or TNFα. Neurons were fixed, permeabilized and labeled with anti-PINCH and stained with phalloidin, the dye that stains actin filaments. Representative confocal images show depolymerization of actin in human neurons exposed to Tat/TNFα. **c** and **d** Quantification of the PINCH fluorescence (**c**) and the actin filament length (**d**). **e** Representative Western blot for lysates from neurons untreated or exposed to Tat or TNFα and probed with antibodies against phospho-Cofilin, Cofilin, Chronophin, and GAPDH. **f** and **g** Quantification of relative protein abundance of phospho-Cofilin/Cofilin (**f**) and Chronophin/GAPDH (**g**) from (**e**). **h** Cell lysates from control, Tat- or TNFα-treated neurons were immunoprecipitated with anti-Parvin antibody. Following immunoprecipitation, total cell lysates (input) and immunoprecipitated materials (IP) were subjected to Western analysis. Samples were probed with antibodies specific for Parvin, TESK1, and ILK1. **i** Schematic representation of PIP complex regulation of cofilin phosphorylation and actin depolymerization. Data represent mean ± SEM; **P < 0.01; ***P < 0.001; *n* = 3–5 (one-way ANOVA)

Given the pivotal role of the PIP complex in regulating cell adhesion, cytoarchitecture and cell survival [46], we assessed if increased PINCH expression alters PIP complex formation and cytoskeletal arrangement that could likely contribute to neurodegeneration. Immunoprecipitation of PINCH indicated that ILK co-immunoprecipition in the presence and absence of Tat/TNFα, whereas α-Parvin interaction was disrupted in neurons exposed to Tat/TNFα (Fig. 2a). As the PIP complex, particularly α-Parvin, provides crucial links between integrins and the actin cytoskeleton, we evaluated actin organization in neurons exposed to Tat or TNFα via immunolabeling for PINCH and F-actin filaments. Results showed disruption of the F-actin filaments in neurons exposed to Tat/TNFα compared to the untreated neurons (Fig. 2b-d).

To investigate potential mechanisms underlying changes in actin organization, we assessed the phosphorylation levels of the actin-binding protein cofilin, which facilitates actin assembly/disassembly. The decreased levels of cofilin phosphorylation at serine 3 in neurons exposed to Tat or TNFα (Fig. 2e, f) indicated that increased cofilin/actin interaction likely promotes actin disassembly. In this context, TESK1 phosphorylates cofilin at serine 3 to block cofilin-mediated actin disruption. Importantly, α-Parvin binding to TESK1 inhibits its kinase activity, thereby promoting cofilin-mediated actin depolymerization. Thus, since α-Parvin dissociated from the PIP complex upon increased PINCH expression (Fig. 2a), we assessed changes in the interactions of α-Parvin with TESK1. In line with our data showing that α-Parvin/ILK interactions decreased with Tat or TNFα, α-Parvin/TESK1 interactions increased (Fig. 2h), further supporting the involvement of cofilin in actin disassembly. The expression levels of TESK1 or chronophin, a phosphatase associated with cofilin, did not change in Tat- or TNFα-treated neurons (Fig. 2a, e, g).

### Increased PINCH expression and actin disassembly are accompanied by perinuclear localization of mitochondria

**Fig. 3:**
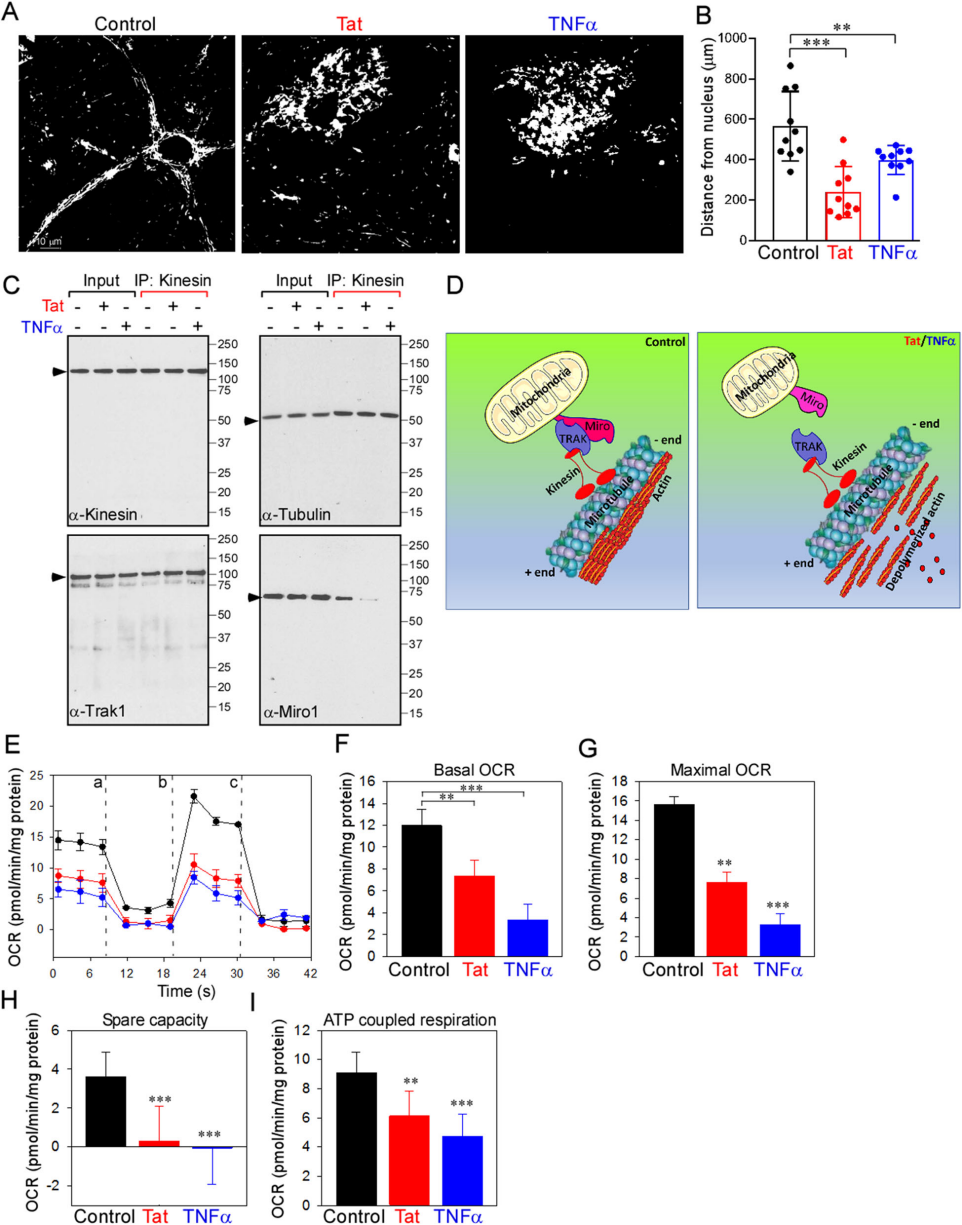
Increased PINCH expression and actin disassembly are accompanied by perinuclear localization of mitochondria. **a** Neurons untreated or exposed to Tat or TNFα for 48 h were monitored for cellular mitochondrial distribution using confocal microscopy. Neurons were stained with dihydrorhodamine (DHR123) and changes in mitochondrial distribution were observed. Representative confocal images show perinuclear localization of mitochondria in neurons exposed to Tat or TNFα. **b** Quantification of the distance (μm) of mitochondria from the nucleus. **c** Cell lysates from control, Tat- or TNFα-treated neurons were immunoprecipitated with antibodies against kinesin. Following immunoprecipitation, total cell lysates (input) and immunoprecipitated materials (IP) were subjected to Western analysis. Samples were probed with antibodies specific for Kinesin (top left), Tubulin (top right), Trak1 (bottom left), and Miro1 (bottom right). **d** Schematic representation shows the disruption of Kinesin-Trak-Miro complex in neurons exposed to Tat or TNFα. **e-i** Neurons were untreated or exposed to Tat or TNFα for 48 h and oxygen consumption rate (OCR) was measured. Untreated neurons were used as control. After measurement of baseline OCR, neurons were sequentially exposed to oligomycin (**a**), carbonyl cyanide-4-(trifluoromethoxy)phenylhydrazone (FCCP) (b), rotenone and antimycin A (**c**). Representative traces of OCR in control and neurons exposed to Tat/TNFα (**e**). Quantification basal (**f**), maximal (**g**), spare capacity (**h**), and ATP coupled respiration (**i**) in control and neurons treated with Tat/TNFα. Data represent mean ± SEM; **P < 0.01; ***P < 0.001; *n* = 3–5 (one-way ANOVA)

Given the actin reorganization observed in neurons exposed to Tat or TNFα (Fig. 2b, c), we examined mitochondrial distribution in control and Tat- or TNFα-treated neurons. Confocal images of neurons exposed to Tat or TNFα labeled with Rhodamine-123 showed aggregation of mitochondria, often clustered around the nucleus, unlike mitochondria in control neurons that were evenly distributed throughout the neuronal soma and processes (Fig. 3a, b). Taken together, these data suggest that neurons exposed to the inflammatory factor TNFα robustly express PINCH, thereby contributing to disruption of the PIP complex, actin disassembly and mitochondrial mislocalization (Supplementary Fig. S2).

To understand how disruption of actin may contribute to mitochondrial mislocalization, we assessed changes in interactions among Tubulin, Kinesin, Trak and Miro1 (Fig. 3c), the protein complex that connects mitochondria to the cytoskeleton (Fig. 3d). Co-immunoprecipitation and Western blot analyses indicated that exposure of neurons to Tat or TNFα disrupted the interaction between Trak and Miro1, while the interactions between Trak and Kinesin, and between Kinesin and Tubulin were preserved (Fig. 3c, d). These data suggest that actin depolymerization contributes to mislocalization of mitochondria by disrupting the Trak/Miro1 interaction (Fig. 3c, d). To assess the bioenergetic capacity of mislocalized mitochondria in neurons exposed to Tat or TNFα, we assessed OCR in all conditions. Neurons exposed to Tat or TNFα were bioenergetically impaired as evidenced by reduced OCR (Fig. 3e-g), decreased spare capacity (Fig. 3h) and diminished ATP-coupled respiration (Fig. 3i).

### Exogenous of PINCH mimics the effects of tat or TNFα, whereas knockdown preserves mitochondrial localization and function

**Fig. 4:**
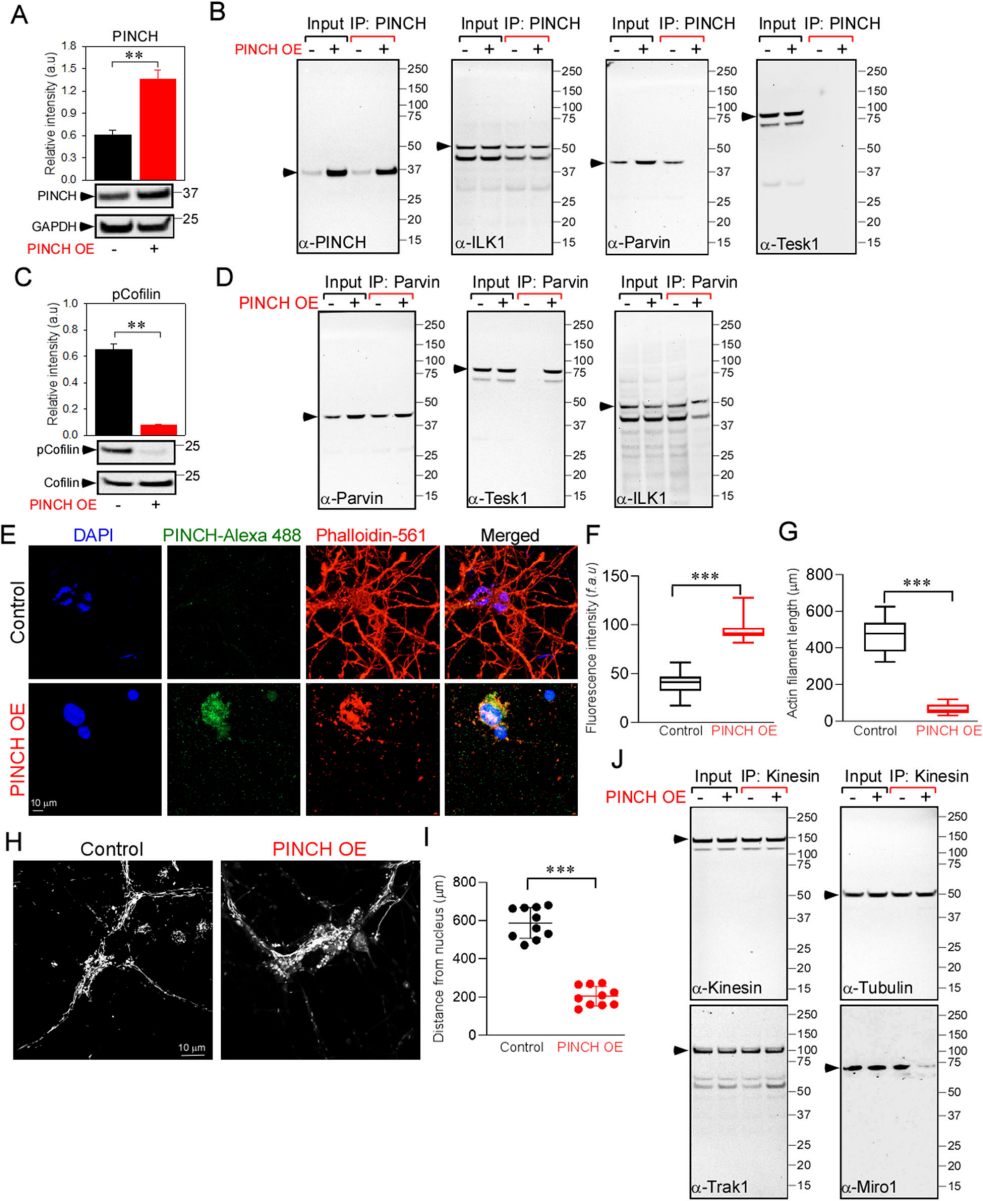
Exogenous expression of PINCH mimics the effects of Tat or TNFα exposure in neurons. **a** Quantification of relative protein abundance (PINCH/GAPDH) and representative Western blots for lysates from neurons with or without exogenous PINCH expression. **b** Cell lysates from control and PINCH-overexpressing (OE) neurons were immunoprecipitated with antibody specific for PINCH. Following immunoprecipitation, total cell lysates (input) and immunoprecipitated materials (IP) were subjected to Western analysis. Samples were probed with antibodies specific for PINCH, ILK1, α-Parvin, and TESK1. **c** Quantification of relative protein abundance (phospho-Cofilin/Cofilin) and representative Western blots for lysates from neurons with or without exogenous PINCH OE. **d** Cell lysates from neurons with or without exogenous PINCH OE were immunoprecipitated with antibody specific for Parvin. Following immunoprecipitation, total cell lysates (input) and immunoprecipitated materials (IP) were subjected to Western blot analysis using antibodies specific for Parvin, TESK, and ILK1. **e** Changes in cellular actin cytoarchitecture were observed using confocal microscopy in neurons with or without exogenous PINCH OE. Neurons were fixed, permeabilized and stained with anti-PINCH and phalloidin, the dye that stains actin filaments. Representative confocal images show depolymerization of actin in neurons exogenously expressing PINCH. (**f** and **g**) Quantification of PINCH fluorescence (**f**) and actin filament length (**g**). **h** Cellular mitochondrial distribution was observed in neurons with or without PINCH expression using confocal microscopy. Neurons were stained with dihydrorhodamine (DHR123) and changes in mitochondrial distribution were observed. Representative confocal images show perinuclear localization of mitochondria in neurons exogenously expressing PINCH. (**i**) Quantification of the distance of mitochondria from the nucleus. **j** Cell lysates from neurons with or without exogenous PINCH expression were immunoprecipitated with antibodies specific for kinesin. Following immunoprecipitation, total cell lysates (input) and immunoprecipitated materials (IP) were subjected to Western blot using antibodies specific for kinesin, Tubulin, Trak1, and Miro1. Data represent mean ± SEM; ***P < 0.001; *n* = 3–5 (one-way ANOVA)

**Fig. 5:**
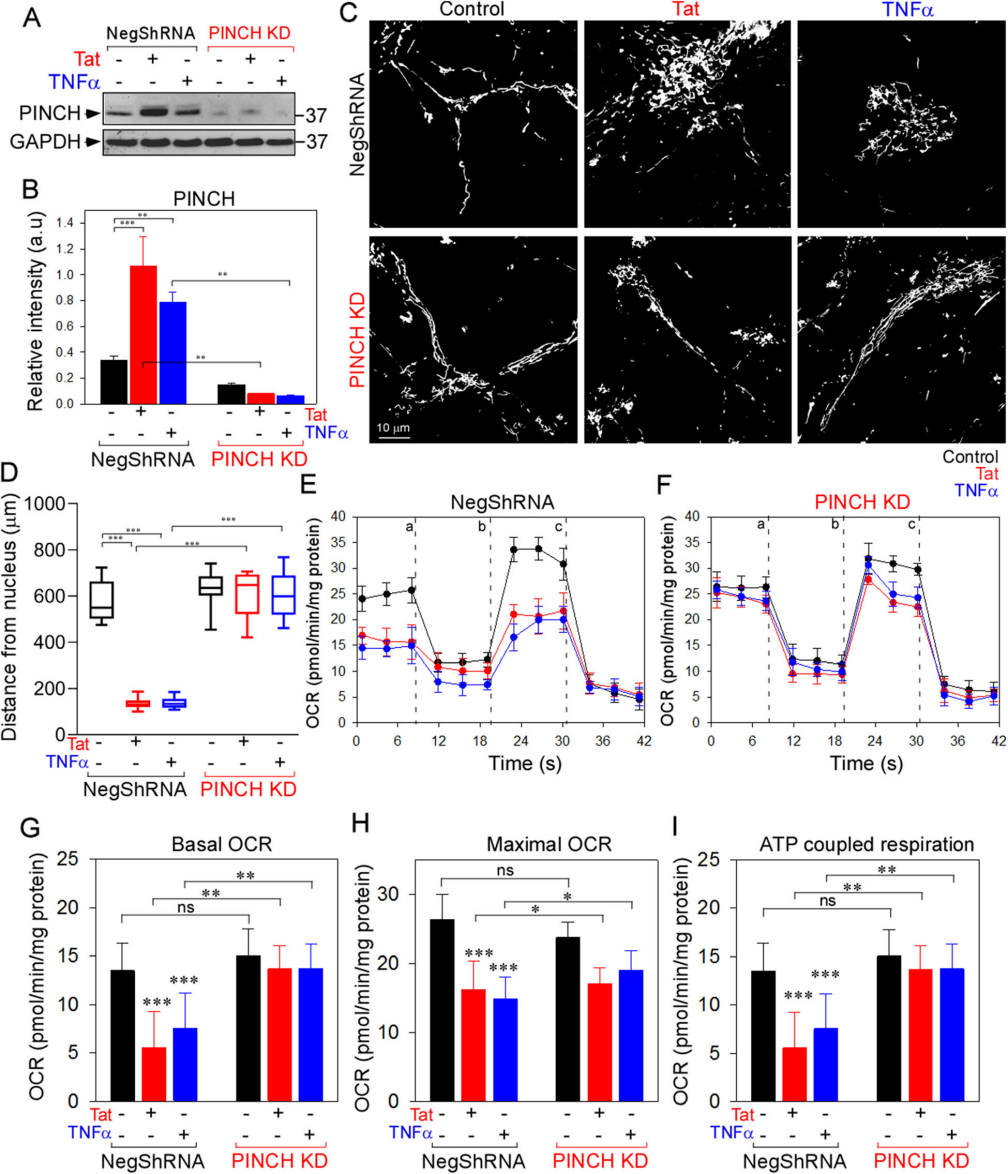
Knock down of PINCH expression preserves neuronal mitochondrial distribution. **a** Western blot analysis showing knock down of PINCH in neurons infected with PINCH shRNA viral particles. **b** Densitometric quantification of PINCH levels in neurons. **c** Mitochondrial morphology was observed in non-targeting shRNA and PINCH KD neurons with or without treatment of Tat or TNFα for 48 h using confocal microscopy. Tat- or TNFα-treated neurons were stained with dihydrorhodamine (DHR123) and changes in mitochondrial morphology were observed. Representative confocal images show preserved mitochondrial distribution in PINCH KD neurons exposed to Tat or TNFα. **d** Quantification of the distance of mitochondria from the nucleus. **e** and **f** Representative traces of OCR in non-target shRNA (**e**) and PINCH KD (**f**) neurons. **g-i** Quantification of basal (**g**), maximal (**h**), and ATP-coupled respiration (**i**) in non-targeting shRNA and PINCH KD neurons exposed to Tat or TNFα

To confirm that increased PINCH expression is ultimately responsible for actin depolymerization, mitochondrial mislocalization and impaired neuronal bioenergetics, we (i) exogenously overexpressed PINCH (Fig. 4), or (ii) knocked down PINCH (Fig. 5), or (iii) knocked down Cav3.1 (Supplementary Fig. S2). Exogenous expression of PINCH in neurons (Fig. 4a) disrupted the PIP complex (Fig. 4b), and reduced the phosphorylation levels of cofilin (Fig. 4c) by increasing the interaction of Parvin with TESK1 (Fig. 4d), thus inactivating TESK1 and promoting cofilin-mediated disruption of actin (Fig. 4e−g). Depolymerization of actin was accompanied by mitochondrial mislocalization (Fig. 4h and i) and disruption of the kinesin-Trak-Miro complex (Fig. 4j). On the other hand, knockdown of PINCH in neurons exposed to Tat or TNFα showed normal distribution of mitochondria (Fig. 5a-c), while in neurons infected with non-targeting shRNA, Tat and TNFα exposure induced mitochondrial mislocalization to the neuronal soma (Fig. 5a-c). Likewise, PINCH KD also prevented the Tat- and TNFα-induced reduction in OCR and ATP-coupled respiration (Fig. 5e-i). As we observed that Cav3.1 KD in neurons prevented Tat- or TNFα-mediated induction of PINCH (Supplementary Fig. S1C, G), we next assessed the effect of Cav3.1 KD on actin and mitochondrial distribution in neurons treated with Tat or TNFα. Cav3.1 KD restored actin polymerization (Supplementary Fig. S3A-C) by increasing cofilin phosphorylation (Supplementary Fig. S3D). In addition, the Tat/TNFα-mediated disruption of the kinesin-Trak-Miro complex (Supplementary Fig. S3E) and mitochondrial mislocalization (Supplementary Fig. S3F, G) were prevented by Cav3.1 KD.

In summary, the present study identified regulatory mechanisms and biological consequences of increased PINCH expression during neuroinflammation. We have shown PINCH expression to be transcriptionally regulated through a Ca^2+^-dependent kinase cascade and that increased PINCH is involved in the disruption of the PIP complex, actin depolymerization, mitochondrial mislocalization to the neuronal soma via disruption of the kinesin-Trak-Miro complex, and bioenergetic crisis. Targeting PINCH expression to maintain baseline levels be a potential new therapeutic strategy for the treatment of neurodegeneration.

## Discussion

PINCH protein is expressed at high levels during development while being nearly undetectable in healthy adult brains or in normal mature neurons. However, previous studies have reported a dramatic increase in PINCH expression in the brains of patients with HIV infection, AD, and epilepsy, and in neurons exposed to the HIV protein Tat or to TNFα [1–3]. Although the expression of PINCH is observed in several inflammatory CNS diseases, the regulatory mechanisms involved in its expression were unknown. Results from our study indicate that PINCH is transcriptionally regulated by MEF2A under inflammatory conditions. The Tat-induced increase in TNFα was observed at 2 h with an approximate ~ 18-fold increase, which was roughly maintained for up to 24 h. However, by 48 h the TNFα levels had increased by approximately 30-fold above control. Although we and others have shown by ELISA that Tat induces TNFα release, early studies reported that Tat induced TNFα and β production through Tat-mediated activation of the TNF-promoter in T-cells and T-cell lines beginning at 8 h and increasing slightly at 16 h and 24 h [47]. Other studies support these findings in monocytes, macrophages, and T-cells [48–51], and also suggest a role of calcium in the induction of TNFα [52]. Studies by Leghmari et al. show that in a calcium-free environment, the Tat-mediated TNFα production was inhibited; whereas, the Tat-mediated IL-10 production remained intact and involved downstream activation of p38 MAPK [53], indicating a potential overlap with Tat and p38 in IL-10 production, independent of TNFα. It is important to assess if TNFα mRNA increases in neurons exposed to Tat, as these cells are not infected by HIV and would provide information as to whether the mRNA dynamics in neurons is similar to those in cells that support HIV infection. Moreover, given that TNFα is just one of the inflammatory factors associated with HIV infection of the CNS, the overlapping roles of Tat-mediated induction of other factors in the context of PINCH production should be examined. Findings from our study support a role of TNFα and Tat-mediated TNFα production in the neuronal changes observed. In this study, we utilized neurons alone to assess these changes. While this approach allows for investigation of neuron-specific signaling changes, other CNS cell interactions were not considered. Cellular crosstalk among neurons, astrocytes, microglia etc., would likely influence these findings. In addition, in the brains of people with HIV (PWH), the levels of neuroinflammation and viral reservoir vary within and among individuals. Another limitation is that the concentration of Tat that we utilized was based on numerous previous in vitro studies [1, 38, 54–61]. Measuring the levels of Tat in the brain has been a challenge, due first to the lack of good antibodies and second to the instability and high rate of degradation of Tat. While assessment of Tat in in vitro studies using overexpression of Tat is easier perform due to the high levels of Tat produced, these levels may not represent physiological levels in PWH with chronic infection.

PINCH is increased in other neurodegenerative diseases besides HIV, making it clear that Tat is not the only factor for the induction of PINCH, but rather likely works in part through induction of TNFα. In this context, Tat and TNF-α signaling are linked in several ways: (1) Tat can induce TNF-α production, (2) the cytotoxic effects of Tat and TNF-α are both synergistic and additive, (3) both Tat and TNF-α induce hpTau at residues commonly observed in tauopathy, and (4) Tat and TNF-α impact integrin signaling and increase PINCH levels. cAMP signaling is known to be increased during HIV-1 infection [62–64] or by TNFα stimulation [65]. A number of functions of prtien kinase A (PKA) during HIV infection have been described, such as the PKA-dependent increase of viral replication and phosphorylation of the HIV proteins p24 and Nef [64, 66, 67]. In this regard, we anticipate that PKA phosphorylates Cav3.1 to increase its activity, as demonstrated in a previous study [68].

ChIP and luciferase assays validate MEF2A as the transcription factor that binds to PINCH promoter to upregulate its expression. In addition, previous studies have shown p38 to be a potent kinase that activates MEF2A [40–42]. Knockdown of Cav3.1 to block the Ca^2+^ entry into cells induced decreased PINCH expression upon decreased MEF2A and p38 phosphorylation, further supporting that MEF2A and p38 are upstream molecules in the signaling cascade. Even though our data suggest that MEF2A and p38K are activated upstream of PINCH, further studies are required to confirm that these factors are required for Tat and TNFα induction of PINCH. MEF2A knockdown or p38K inhibition are needed to explore the possible contribution of other pathways.

Our data also suggest that elevated cCa^2+^ activates MEF2A through p38 phosphorylation and facilitates PINCH expression, however, additional pathways may be involved. For example, the T-type channels are thought to pump Ca^2+^ directly into the mitochondria/ER [69, 70], while cCa^2+^ is not affected. To distinguish between the two compartments, future studies should address Ca^2+^ flux into mitochondria using mitochondrial-specific Ca^2+^ indicators. For example, if mitochondrial Ca^2+^ is increased, this could be another source of damage to mitochondrial functioning and possibly contribute to the observed effects of TNFα and Tat on respiration, morphology and distribution. In this context, upon slight depolarization of the cell and in the absence of action potentials, a sustained entry of Ca^2+^ within a permissive window of voltage is possible due to the significant overlap between the activation and inactivation potential ranges of the channel [71–73]. Although this steady-state current is barely detectable with conventional patch-clamp techniques, it is sustained and allows a significant amount of Ca^2+^ to enter and accumulate within the cell. Moreover, the resting membrane potential of neurons has been estimated to be approximately 70 mV and as a consequence, a slight depolarization of the membrane may increase the steady-state influx of Ca^2+^ through T channels [74]. In this regard, we observed increased cCa^2+^ in neurons exposed to Tat or TNFα was absent when Cav3.1 was knocked down.

The dynamic organization of the actin cytoskeleton is crucial for numerous cell processes [75]. For example, actin waves from the cell body to the tip of the neurites generate a flow that transiently widens the neurite shafts. Stochastically, this creates the space needed for microtubules to polymerize and create tracks for kinesin-based transport of cargoes/organelles [76]. Mitochondria are one such organelle that travel along the cytoskeleton using microtubules for long-distance trafficking to meet the high energy demands of neurons by supplying ATP. However, the regulatory connections between actin and tubulin for proper mitochondrial distribution have only been recently reported [77–81]. Kinesin is essential for the anterograde mitochondrial transport via an adaptor complex of kinesin-Trak-Miro proteins [82, 83] with Trak forming a bridge between kinesin and Miro1. PINCH is nearly undetectable in healthy adult brains [3, 4] but is induced in CNS diseases and in cell and animal models of neurodegeneration. Our studies published in 2008 showed for the first time that PINCH was robustly detected in the brains and cerebrospinal fluid (CSF) of HIV patients [4]. Importantly, our earlier studies reported an increase in levels of hpTau in in vitro neuronal cultures where PINCH was overexpressed or induced by exposure to TNFα [1, 3]. In fact, blocking PINCH expression with shRNA in neurons exposed to hpTau-inducing agents decreased the levels of hpTau [3]. Likewise, in a tauopathy mouse model, PINCH was shown to be significantly increased in several brain regions, where it was undetectable in wildtype mice [3]. Our findings were then expanded to the brains of AD, frontotemporal dementia and mesial temporal epilepsy patients [2, 3]. Until recently, the overlap of increased PINCH and hpTau levels was unclear. Our data indicate that along with actin depolarization and mitochondrial mislocalization, microtubule interactions with various protein components (Miro1, Trak, kinesin) are altered. In this context, it is possible that these disruptions also contribute abnormal Tau hyperphosphorylation and dissociation from the tubulin cytoskeleton. Understanding these potential interactions warrants further investigation into how alterations in Tublin, Trak, miro1 and kinesin impact Tau.

Our results indicate that increased PINCH expression is accompanied by disruption of the PIP complex and actin disassembly. The cytosolic PIP complex can promote actin cytoskeletal rearrangement and growth factor signaling through interactions with Nck2 [8, 84, 85]. Forced disruption of the PIP complex by overexpression of the PINCH-binding domain of ILK was reported to decrease cell-matrix adhesion and induce apoptosis in podocytes [46, 86]. Increased PINCH expression and actin disassembly are accompanied by perinuclear localization of mitochondria. The mislocalization of mitochondria may be caused directly by the PIP or actin disruption, or other mechanism(s) may be involved. For example, studies by De Vos et al. showed that the TNFα-induced hyperphosphorylation of kinesin light chain inhibited the kinesin-mediated transport of mitochondria [87]. Given that there are several isoforms of kinesin that are differentially phosphorylated by TNFα, direct effects of PIP disruption and actin disassembly on mitochondrial mislocalization may not be the only factors involved. Here, we examined the heavy-chain kinesin KIF5B that has been shown to regulate mitochondrial network changes [88] and showed that the increased PINCH expression caused KIF5B to dissociate from Miro, which provided evidence that this mechanism does contribute at least in part to mitochondrial mislocalization.

Our results proposing that some components of HIV contribute to mislocalization of mitochondria in neurons are consistent with several other studies [89–93]. In these previous studies, Tat and gp120 are reported to decrease mitochondrial membrane potential and recruit mitophagy markers to damaged mitochondria, but impair the delivery of mitochondria to the lysosomal compartment, causing incomplete mitophagy [93]. Other studies described elongated mitochondria in neurons exposed to Tat or gp120 via viral protein-mediated changes in levels of mitochondrial fission and fusion proteins [89, 90, 92]. Exogenous PINCH mimics the effects of Tat or TNFα, whereas PINCH knockdown preserves mitochondrial localization and function. Since our previous studies indicate that Tat and TNFα, but not gp120, induce PINCH expression [1], data presented in this manuscript show that PINCH expression is involved in the mitochondrial changes observed. We cannot rule out that TNFα or Tat may not independently affect mitochondrial morphology and this should be explored in the context of PINCH knockdown in the presence of Tat, TNFα and possibly gp120.

## Conclusions

Given the lack of mechanistic links among PINCH, neuroinflammation, neurodegeneration and mitochondrial dysfunction, our study is the first to uncover an important regulatory mechanism for PINCH expression and some of the neuropathological consequences of increased PINCH in neurons. We have identified the transcription factor responsible for PINCH induction in TNFα-mediated neuroinflammatory conditions and the biological consequences of increased PINCH expression in neurons. Given that AD and neuroHIV share some pathological features including cognitive impairment with chronic neuroinflammation, Tau pathology, and robust neuronal expression of PINCH, the present study provides information for new targetable pathways to lessen bioenergetic deficits observed in the brains of PWH and other neurogenerative disorders. Further studies on potential strategies to inhibit PINCH induction during neuroinflammatory disorders are warranted. Several lines of investigation into these approaches are underway, including strategies to maintain PINCH at normal levels at both transcriptional and translational levels utilizing TNF-receptor and calcium influx antagonists, and a newly-discovered miRNA involved in PINCH translational regulation. The goal of these studies is to drive PINCH expression to physiological levels to alleviate bioenergetic deficits and cytoskeletal disruptions observed in chronic neuroinflammatory diseases.

## Supplementary information

**Additional file 1: Supplementary Figure S1.** Elevated cytosolic Ca^2+^ activates MEF2A through P38 phosphorylation and facilitates PINCH expression. (A) Neurons untreated or exposed to Tat or TNFα for 48 h were loaded with Fluo-4 AM (5 μM) to measure cytosolic Ca^2+^ levels. Quantification of Fluo-4 fluorescence at baseline levels. Three independent experiments were performed. Each dot represents mean fluorescence of ~ 10 cells/field and 5 fields/experiment were quantified. (B) Scr siRNA and Cav3.1 KD human neurons untreated or exposed to Tat or TNFα for 48 h were loaded with Fluo-4 AM (5 μM) to measure cytosolic Ca^2+^ levels. Fluo-4 fluorescence quantified as in (A). (C) Representative Western blots for lysates from Scr siRNA and Cav3.1 KD neurons untreated or exposed to Tat or TNFα for 48 h and probed with antibodies against Cav3.1, phospho-P38, P38, phospho-MEF2A, MEF2A, PINCH and GAPDH. (D-G) Quantification of relative protein abundance of Cav3.1 (D), phospho-P38 (E), phospho-MEF2A (F) and PINCH (G) from (C). (H) ChIP-assay was performed in Scr siRNA and Cav3.1 KD neurons untreated or exposed to Tat or TNFα for 48 h. Anti-MEF2A antibody was used to immunoprecipitate the chromatin and the fold enrichment of *lims1/pinch* promoter relative to the matched input control was quantified by q-PCR. (I) Luciferase activity was measured in Scr siRNA and Cav3.1 KD neurons transfected with *lims1/pinch* luciferase construct after treatment with or without Tat or TNFα for 48 h. Data represent mean ± SEM; **P < 0.01; ***P < 0.001; *n* = 3–5 (one-way ANOVA).

**Additional file 2: Supplementary Figure S2.** Schematic representation of HIV-Tat/TNFα-mediated PINCH expression and its associated signaling pathways. cAMP levels are known to be increased during HIV infection or TNFα treatment. The increased cAMP levels lead to increased protein kinase A (PKA) activity which in turn phosphorylates Cav3.1 and increases its activity. The dotted blue line depicts cAMP<PKA < Cav3.1 activation based on previous literature. The activation of Cav3.1 increases the entry of Ca^2+^ into cells. The increased cytosolic Ca^2+^ increases expression of PINCH through the CAMKII<p38 < MEF2A pathway. The solid green lines depict the data from our current study. Increased expression of PINCH triggers the release of Parvin from the PINCH-ILK-Parvin (PIP complex) complex. Released Parvin interacts with Tesk1, inhibiting cofilin phosphorylation and activates actin disruption.

**Additional file 3: Supplementary Figure S3.** Cav3.1 KD blocks PINCH induction and preserves neuronal mitochondrial distribution. (A) Cellular actin architecture in Scr siRNA and Cav3.1 knockdown (KD) neurons untreated or exposed to Tat or TNFα for 48 h using confocal microscopy. Neurons were fixed, permeabilized and labeled with anti-PINCH and stained with phalloidin. Representative confocal images show preserved actin polymerization in Cav3.1 KD neurons exposed to Tat or TNFα. (B and C) Quantification of the PINCH fluorescence (B) and the actin filament length (C). (D) Quantification of relative protein abundance (phospho-Cofilin/Cofilin) and representative Western blots of lysates from Scr siRNA and Cav3.1 KD human neurons untreated or exposed to Tat or TNFα. (E) Cell lysates from Scr siRNA and Cav3.1 KD neurons untreated or exposed to Tat or TNFα were immunoprecipitated with anti-kinesin antibody. Following immunoprecipitation, total cell lysates (input; left) and immunoprecipitated materials (IP; right) were subjected to Western blot analysis. Samples were probed with antibodies against Tubulin, kinesin and Miro1. (F) Mitochondrial distribution was observed in Scr siRNA and Cav3.1 KD neurons untreated or exposed to Tat or TNFα for 48 h using confocal microscopy. Neurons were stained with dihydrorhodamine (DHR123) and changes in mitochondrial distribution were observed. Representative confocal images show preserved mitochondrial distribution in Cav3.1 KD neurons exposed to Tat or TNFα. (G) Quantification of the distance (μm) of mitochondria from the nucleus. Data represent mean ± SEM; ***P < 0.001; *n* = 3–5 (one-way ANOVA).

## Data Availability

Data sharing is not applicable to this article as no datasets were generated or analyzed during the current study.

## Abbreviations

AD: Alzheimer’s disease
ANK: Ankyrin
CAMKII: Calcium/calmodulin-dependent protein kinase II
Cav3.1: Low-voltage-activated CaV3.1 calcium channel
cCa^2+^: Cytosolic calcium
ChIP: Chromatin immunoprecipitation
CNS: Central nervous system
ECM: Extracellular matrix
FDU: 5-fluoro-2′-deoxyuridine
GFAP: Glial fibrillary associated protein
HBSS: Hank’s balanced salt solution
HIV: Human immunodeficiency virus
hpTau: Hyperphosphorylated Tau
ILK: Integrin linked kinase
KD: Knockdown
LatA: Latrunculin A
*lims1*: LIM zinc finger domain containing 1 gene
MAP2: Microtubule associated protein 2
MEF2A: Myocyte enhancer factor 2A
NM0: Neurobasal media
PCR: Polymerase chain reaction
PWH: People with HIV
PINCH: Particularly interesting new cysteine histidine-rich protein
PIP: PINCH-ILK-Parvin
Tat: Transactivator of transcription
TESK1: Testis associated actin remodeling kinase 1
TNFα: Tumor necrosis factor alpha
TGF-β1: Transforming growth factor beta 1
