# Supplementary figures and images for: Inflammation-induced PINCH expression leads to actin depolymerization and mitochondrial mislocalization in neurons
Source: Transl Neurodegener. 2020 Aug 3;9:32. doi: 10.1186/s40035-020-00211-4 (PMC7397656; doi:10.1186/s40035-020-00211-4)

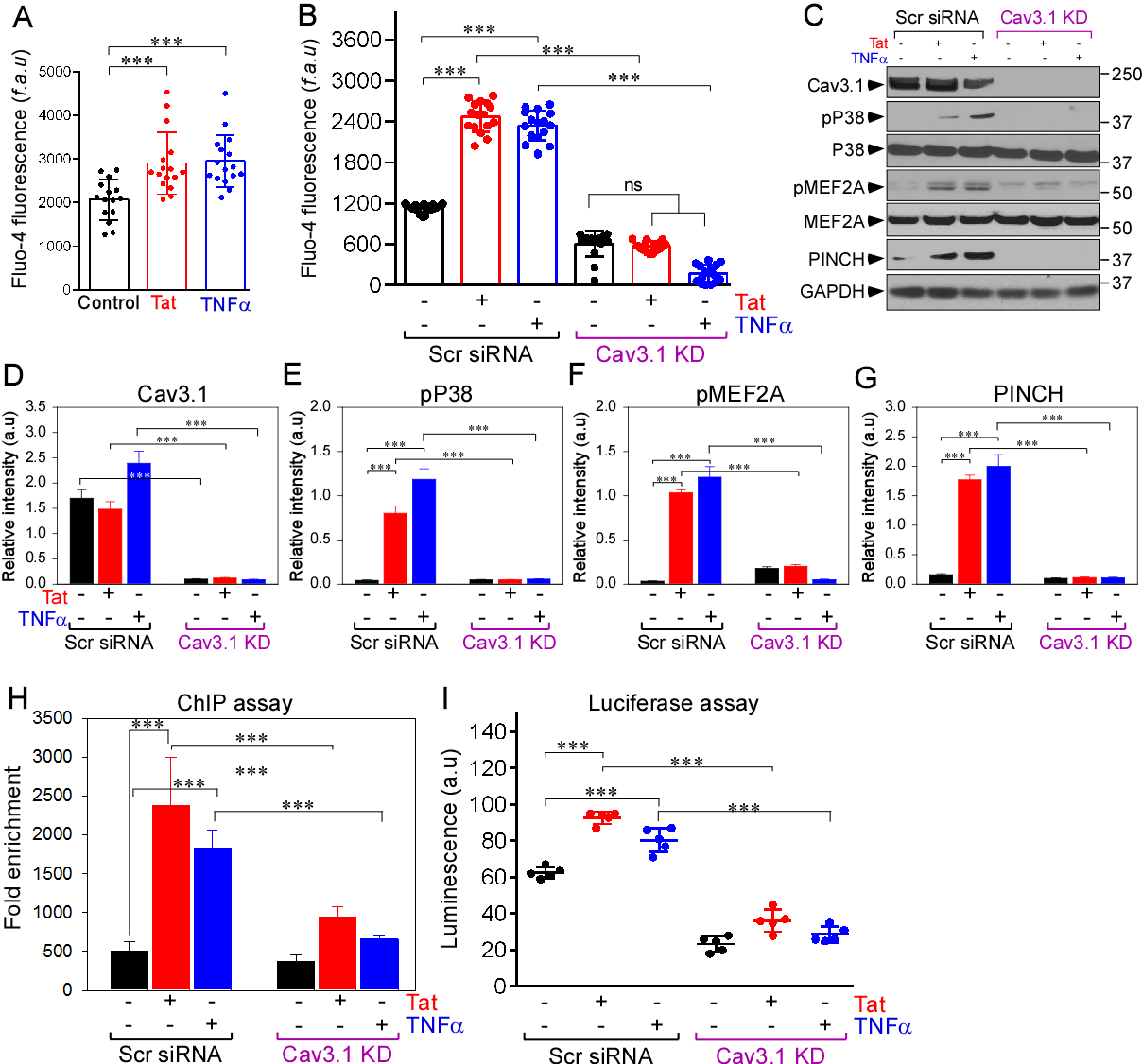

Supplement: Supplementary file 1 — Additional file 1: Supplementary Figure S1. Elevated cytosolic Ca2+ activates MEF2A through P38 phosphorylation and facilitates PINCH expression. (A) Neurons untreated or exposed to Tat or TNFα for 48 h were loaded with Fluo-4 AM (5 μM) to measure cytosolic Ca2+ levels. Quantification of Fluo-4 fluorescence at baseline levels. Three independent experiments were performed. Each dot represents mean fluorescence of ~ 10 cells/field and 5 fields/experiment were quantified. (B) Scr siRNA and Cav3.1 KD human neurons untreated or exposed to Tat or TNFα for 48 h were loaded with Fluo-4 AM (5 μM) to measure cytosolic Ca2+ levels. Fluo-4 fluorescence quantified as in (A). (C) Representative Western blots for lysates from Scr siRNA and Cav3.1 KD neurons untreated or exposed to Tat or TNFα for 48 h and probed with antibodies against Cav3.1, phospho-P38, P38, phospho-MEF2A, MEF2A, PINCH and GAPDH. (D-G) Quantification of relative protein abundance of Cav3.1 (D), phospho-P38 (E), phospho-MEF2A (F) and PINCH (G) from (C). (H) ChIP-assay was performed in Scr siRNA and Cav3.1 KD neurons untreated or exposed to Tat or TNFα for 48 h. Anti-MEF2A antibody was used to immunoprecipitate the chromatin and the fold enrichment of lims1/pinch promoter relative to the matched input control was quantified by q-PCR. (I) Luciferase activity was measured in Scr siRNA and Cav3.1 KD neurons transfected with lims1/pinch luciferase construct after treatment with or without Tat or TNFα for 48 h. Data represent mean ± SEM; **P < 0.01; ***P < 0.001; n = 3–5 (one-way ANOVA). [file 40035_2020_211_MOESM1_ESM.tif]

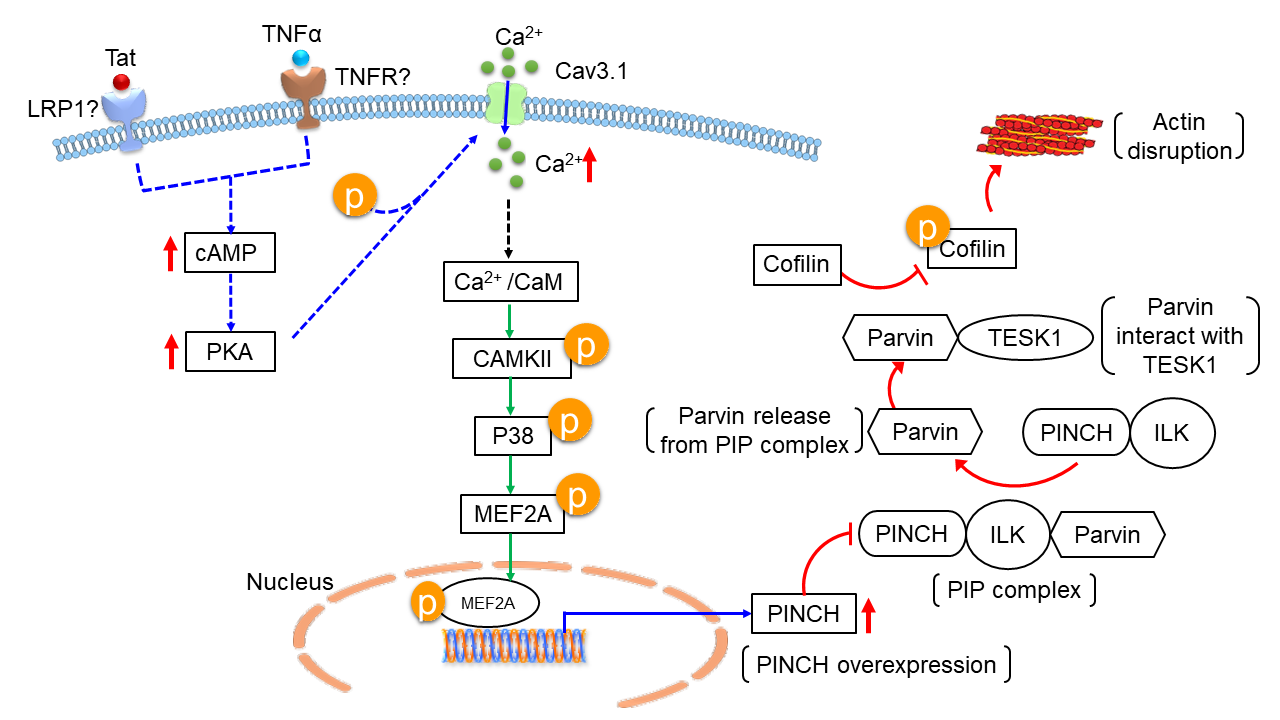

Supplement: Supplementary file 2 — Additional file 2: Supplementary Figure S2. Schematic representation of HIV-Tat/TNFα-mediated PINCH expression and its associated signaling pathways. cAMP levels are known to be increased during HIV infection or TNFα treatment. The increased cAMP levels lead to increased protein kinase A (PKA) activity which in turn phosphorylates Cav3.1 and increases its activity. The dotted blue line depicts cAMP<PKA < Cav3.1 activation based on previous literature. The activation of Cav3.1 increases the entry of Ca2+ into cells. The increased cytosolic Ca2+ increases expression of PINCH through the CAMKII<p38 < MEF2A pathway. The solid green lines depict the data from our current study. Increased expression of PINCH triggers the release of Parvin from the PINCH-ILK-Parvin (PIP complex) complex. Released Parvin interacts with Tesk1, inhibiting cofilin phosphorylation and activates actin disruption. [file 40035_2020_211_MOESM2_ESM.tif]

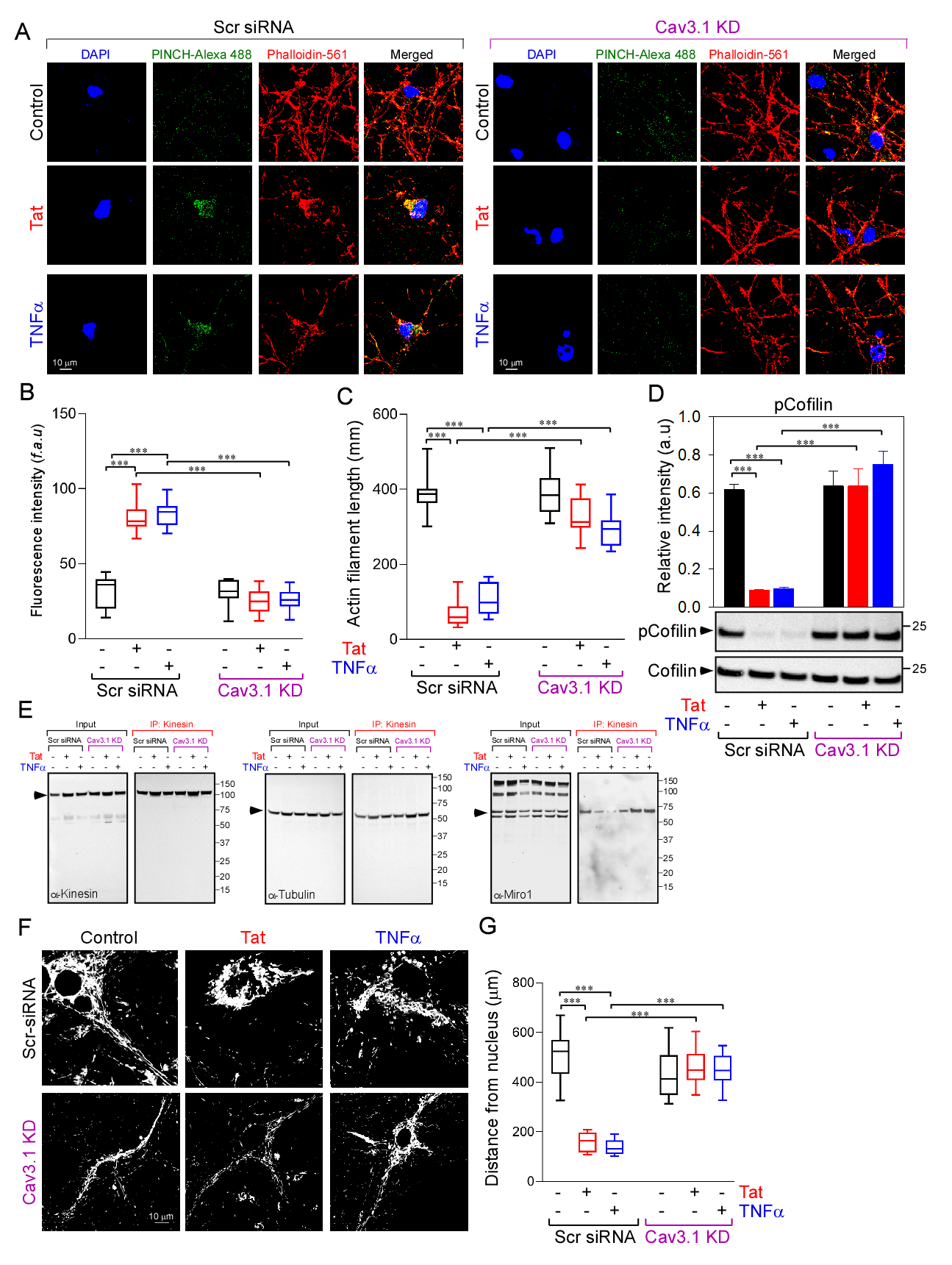

Supplement: Supplementary file 3 — Additional file 3: Supplementary Figure S3. Cav3.1 KD blocks PINCH induction and preserves neuronal mitochondrial distribution. (A) Cellular actin architecture in Scr siRNA and Cav3.1 knockdown (KD) neurons untreated or exposed to Tat or TNFα for 48 h using confocal microscopy. Neurons were fixed, permeabilized and labeled with anti-PINCH and stained with phalloidin. Representative confocal images show preserved actin polymerization in Cav3.1 KD neurons exposed to Tat or TNFα. (B and C) Quantification of the PINCH fluorescence (B) and the actin filament length (C). (D) Quantification of relative protein abundance (phospho-Cofilin/Cofilin) and representative Western blots of lysates from Scr siRNA and Cav3.1 KD human neurons untreated or exposed to Tat or TNFα. (E) Cell lysates from Scr siRNA and Cav3.1 KD neurons untreated or exposed to Tat or TNFα were immunoprecipitated with anti-kinesin antibody. Following immunoprecipitation, total cell lysates (input; left) and immunoprecipitated materials (IP; right) were subjected to Western blot analysis. Samples were probed with antibodies against Tubulin, kinesin and Miro1. (F) Mitochondrial distribution was observed in Scr siRNA and Cav3.1 KD neurons untreated or exposed to Tat or TNFα for 48 h using confocal microscopy. Neurons were stained with dihydrorhodamine (DHR123) and changes in mitochondrial distribution were observed. Representative confocal images show preserved mitochondrial distribution in Cav3.1 KD neurons exposed to Tat or TNFα. (G) Quantification of the distance (μm) of mitochondria from the nucleus. Data represent mean ± SEM; ***P < 0.001; n = 3–5 (one-way ANOVA). [file 40035_2020_211_MOESM3_ESM.tif]
